# Characterization and Radiation Response of Cancer-Associated Fibroblasts isolated from Glioblastoma

**DOI:** 10.17912/micropub.biology.001810

**Published:** 2025-09-26

**Authors:** Caroline Delmas, Elisabeth Cohen-Jonathan-Moyal, Catherine Seva

**Affiliations:** 1 Oncopole Claudius Regaud, IUCT-Oncopole, Toulouse, France; 2 Centre de Recherches en Cancérologie de Toulouse, CRCT, Université de Toulouse, INSERM, CNRS, Toulouse, France.

## Abstract

Glioblastoma (GBM) is an aggressive brain tumor with poor prognosis and strong resistance to radiotherapy. While cancer-associated fibroblasts (CAFs) are known in epithelial cancers, their presence in GBM has only recently been identified. In this study we isolated GBM-derived CAFs and confirmed their identity with specific markers. After irradiation (4–6 Gy), these CAFs showed no morphological or phenotypic changes and maintained viability, demonstrating radioresistance. The results indicate that CAFs may play a key role in GBM’s resistance to treatment, underlining the need for further research on their role in radiotherapy failure.

**Figure 1. Characterization and Radiation Response of Cancer-Associated Fibroblasts isolated from Glioblastoma:**
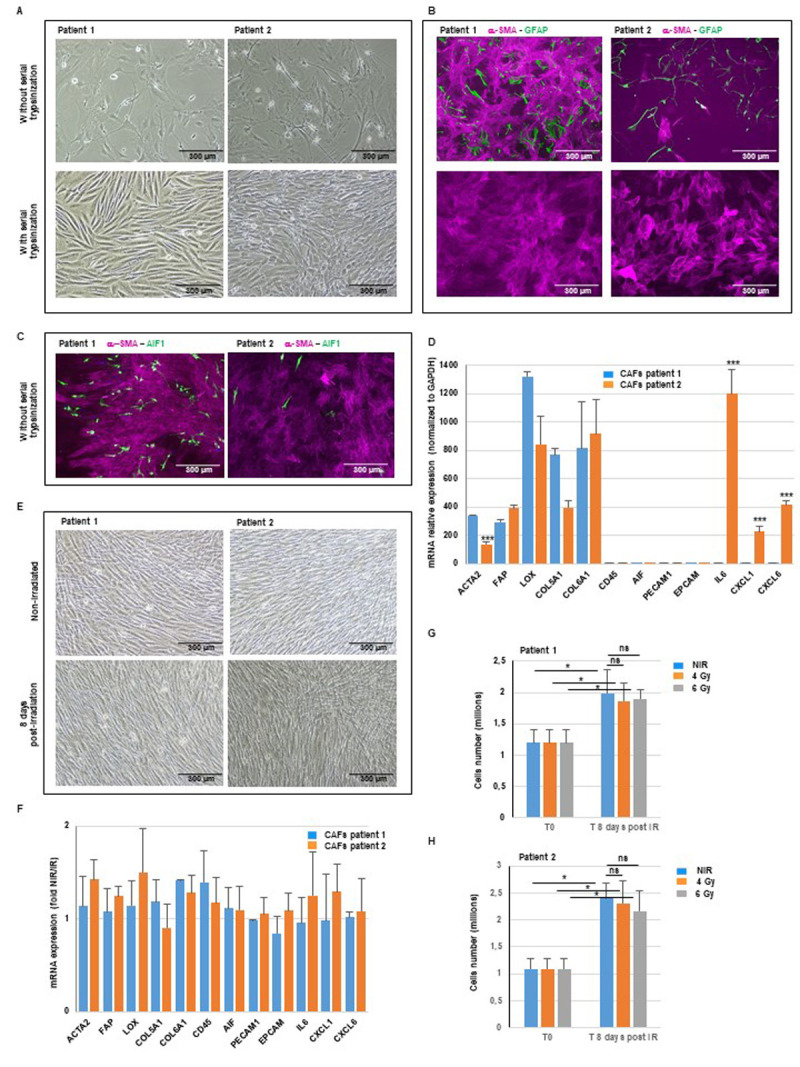
Primary cultures derived from two GBM patients, with or without the application of serial trypsinization, were (A) analyzed under a bright field microscope and (B, C) characterized by immunofluorescence staining for the markers α-SMA, GFAP, and AIF1. Pictures (10X) are representative of at least 3 independent experiments. (D) Following serial trypsinization, CAFs derived from the two GBM patients were analyzed by real-time PCR for the expression of CAFs markers (ACTA2, FAP, LOX, COL5A1, COL6A1), non CAFs markers (CD45, AIF1, PECAM1, EPCAM) and cytokines (IL6, CXCL1, CXCL6). Quantifications of 3 independent experiments each performed in duplicate are presented as means ± SD. P-value, ***P<0.001 (T-test between patient 1 and patient 2). (E) Bright field pictures (10X) of GBM patients-derived CAFs 8 days post-irradiation (6 Gy). Pictures (10X) are representative of at least 3 independent experiments. (F) mRNA of irradiated or non-irradiated CAF (6 Gy) were analyzed by real-time PCR for the CAFs markers and non-CAFs markers. Results are presented as fold changes of irradiated versus non-irradiated CAFs. Quantifications of 3 independent experiments each performed in duplicate are presented as means ± SD. For each gene, t-test P-value between non irradiated and irradiated conditions was non-significant, P>0.05. (G, H) The viability of GBM patients-derived CAFs following the administration of single doses of 4 Gy and 6 Gy was evaluated 8 days post-irradiation by cell counting. Quantifications of 4 independent experiments each performed in duplicate are presented as means ± SD. P-value, ns: P>0.05 (ANOVA between non irradiated and irradiated with doses of 4 or 6 Gy). P-value, *0.01<P<0.05 (ANOVA between T0 and T8 days post irradiation).

## Description

Glioblastoma (GBM) is the most frequently diagnosed brain tumor among adults. It is characterized by its highly invasive nature, leading to diffuse infiltration into brain tissue, which complicates surgical resection and often results in incomplete removal. Furthermore, GBMs exhibit significant resistance to postoperative therapies. Despite the use of combined radiotherapy and chemotherapy, the majority of GBMs tend to recur, with a median survival rate of approximately 15 months (Weller, Butowski et al. 2017).

Besides the inherent resistance demonstrated by GBM cells, the cells present in the microenvironment can significantly influence tumor cells aggressiveness. In the case of GBM, various studies have indicated that endothelial and immune cells play a role in tumor progression by specifically affecting growth and invasion (Aghajani, Jalilzadeh et al. 2024, Hovis, Chandra et al. 2024, Jang and Park 2025). Conversely, there has been minimal focus on the presence and function of cancer-associated fibroblasts (CAFs) in GBM.

CAFs are recognized as a crucial cell type in the stroma of epithelial cancers in which their interaction with cancer cells and other stromal cells can influence a wide range of processes contributing to tumor progression such as invasion, angiogenesis, anti-tumor immunity or even resistance to therapies including irradiation (O'Fee, Burley et al. 2025, Zhang, Wang et al. 2025).

CAFs specifically regulate these functions via cell-cell interactions, as well as by releasing a multitude of factors, including extracellular matrix proteins, chemokines, cytokines, and growth factors.

Historically, it was believed that fibroblasts were absent from the central nervous system, which raised questions about the existence of CAFs in GBM.

Nevertheless, a recent analysis using single-cell RNA sequencing and spatial transcriptomics has revealed the presence of CAFs-like stromal cells in human GBM (Jain, Rick et al. 2023). This cell population exhibits a characteristic CAFs morphology, high expression of several recognized CAFs markers (such as ACTA2, FAP) and lacks the expression of non-CAFs stromal cell markers that share traits with CAFs (such as epithelial, endothelial, pericytes and immune cells). They are found in perivascular and peritumoral niches, frequently in close proximity to vascular structures and areas rich in extracellular matrix (ECM). This report also revealed that CAFs enhance the stemness of GBM cells, promote the pro-tumor M2 phenotype in macrophages, and stimulate tumor growth in vivo through the release of cytokines and growth factors.

Subsequently, two additional studies validated the existence of CAFs in GBM and illustrated their contribution to tumor progression (Zhao, Yang et al. 2023, Galbo, Madsen et al. 2024). These investigations showed in particular that a microenvironment enriched in CAFs correlates with tumor grade and is inversely related to the survival rates of GBM patients. In addition, bioinformatic analyses of GBM transcriptomic databases, demonstrated that GBMs characterized by a high CAFs signature exhibit elevated expression of genes associated with invasion. The findings from this bioinformatics study were corroborated by utilizing conditioned medium from a commercial CAFs line derived from GBM, which resulted in enhanced migration and invasion of GBM cells from patients.

Radiotherapy is a commonly utilized treatment for solid tumors, with over half of cancer patients receiving this therapy. It is established as the standard care for GBM. Nonetheless, the inherent radioresistance of tumor cells, whether intrinsic or induced by radiotherapy (RT), remains a major problem in tumor treatment. Additionally, the tumor microenvironment (TME) is also affected by radiation and can undergo substantial alterations due to RT. CAFs derived from different solid tumors, such as prostate, lung, colorectal, and pancreatic cancers, have demonstrated a notable resistance to radiation, especially compared to normal fibroblasts. Instead of being rendered inactive by RT, they remain as active participants within the TME. Irradiated CAFs persist in secreting factors that contribute to tumor recurrence and resistance to therapy (Zhang, Lv et al. 2023).

To our current knowledge, the radioresistance of CAFs derived from GBM has not been assessed.

In this study, we isolated CAFs by successive trypsinization from GBM patient samples, a method commonly described to purify CAFs in other cancers. We then characterized these CAFs and examined how irradiation affects their phenotype and survival.

Cells were isolated and cultured according to the procedures outlined in the materials and methods section, using fresh samples from two patients diagnosed with GBM. Prior to serial trypsinization, figure 1A illustrates, on these two primary cell cultures, observed under bright field microscopy, a heterogeneous cell population, which includes CAFs, glial cells, and macrophages. The cells subsequently underwent serial trypsinization to eliminate less adherent cells, primarily tumor cells and non-CAFs stromal cells, leading to the retention of a cell population that is resistant to trypsinization and exhibits a fibroblastic morphology, which has been identified as CAFs in other types of cancer. After serial trypsinization (figure 1A), a predominant population displaying the morphology characteristic of CAFs and fibroblasts is observed.

Cellular heterogeneity prior to serial trypsinization was also detected through immunofluorescence utilizing a CAFs-specific marker, α-SMA, a glial cell-specific marker, GFAP, which identifies GBM cells, along with an immune cell marker, AIF1 (figures 1B, 1C). Following serial trypsinization, the CAFs-enriched cell population shows almost exclusive α-SMA staining (figures 1B, 1C).

As emphasized in numerous recent publications, CAFs represent a heterogeneous population with no single marker for their identification (Biffi and Tuveson 2021, Lavie, Ben-Shmuel et al. 2022). Therefore a combination of multiple markers is used to better characterize CAFs. Following serial trypsinization, the CAFs were analyzed by a real-time PCR approach to investigate the expression of these different markers. Figure 1D shows, in the CAFs-enriched cell population, after serial trypsinization, a high expression of various well-known CAFs markers including ACTA2 the gene coding for α-SMA protein , FAP, LOX, and various collagens, alongside the absence of markers related to non-CAFs stromal cells that share common features with CAFs, such as epithelial cells (EPCAM), endothelial cells/pericytes (PECAM1), and immune cells (CD45, AIF1).

In recent years, there has been a growing acknowledgment that the intra-tumoral CAFs population includes several subsets (Biffi and Tuveson 2021, Lavie, Ben-Shmuel et al. 2022, Luo, Xia et al. 2022). While there is no definitive agreement on the exact number of subsets, two subtypes are commonly observed in most analyses. Myofibroblastic CAFs (myCAFs), which are heavily involved in ECM deposition and express high levels of ACTA2, and inflammatory CAFs (iCAFs), which are marked by an elevated production of immunomodulating cytokines and lower levels of ACTA2.

Figure 1D shows that these two CAFs subtypes are found in GBM. Patient 1’s CAFs exhibit characteristics close to myCAFs showing significant elevated levels of ACTA2 in comparison to Patient 2 (T-test ***P<0.001) and no production of IL6, CXCL1, or CXCL6. In contrast, the CAFs from Patient 2 resemble iCAFs, marked by a significant decrease in ACTA2 expression and significantly higher levels of cytokines when compared to Patient 1 (T-test ***P<0.001). As previously stated, the majority of CAFs derived from various solid tumors exhibit resistance to radiation. Nevertheless, the radioresistance of CAFs derived from GBM has not been investigated. Consequently, we assessed the effect of radiation on the survival of GBM-derived CAFs by delivering single doses of 4 Gy and 6 Gy, which are commonly used to determine the radioresistance of GBM cells and GBM stem cells (Lacore, Delmas et al. 2022). As illustrated in Figure 1E, irradiated CAFs exhibit minimal to no morphological alterations when compared to non-irradiated CAFs. Moreover, we did not detect any changes in the phenotype of CAFs following irradiation, as the expression levels of both CAFs-specific markers and non-CAFs markers in figure 1F remained unchanged after exposure to irradiation (T-test P-value between non irradiated and irradiated conditions non-significant P>0.05). The survival rate of the irradiated CAFs was determined through cell counting. In figures 1G and 1 H, we noted a significant increase in cell proliferation between T0 and T8 days post irradiation (ANOVA *0.01<P<0.05). However we observed no significant variations in the proliferation of CAFs at 8 days following irradiation with doses of 4 or 6 Gy, in comparison to non-irradiated CAFs (ANOVA P-value >0.05), which suggests a radioresistance in CAFs.

This study is the first to analyze the radioresistance of CAFs derived from GBM. The results obtained are in agreement with prior reports on the radioresistance of CAFs derived from different solid tumors.

The presence of CAFs following radiotherapy in GBM may represent a significant obstacle to treatment efficacy, similar to what is observed in other cancers. Gaining insight into and addressing these mechanisms will be crucial for enhancing the effectiveness of radiotherapy.

## Methods

***Culture of CAFs derived from GBM biopsy specimens.*** Biopsies of glioblastoma (GBM) were collected from the Department of Neurosurgery at Toulouse University Hospital. This clinical investigation, directed by Professor E. Cohen-Jonathan-Moyal, received authorization from the Human Research Ethics Committee (Ethics Code: NCT06222138, PI Elizabeth Cohen-Jonathan-Moyal). All participants provided written informed consent. Patients characteristics: Patient 1, male 74 years old; patient 2, female 73 years old. Tumor tissue samples that were surgically resected before radio-chemotherapy were mechanically dissociated and filtered on 100µm filters then incubated in 6-well dishes containing DMEM-F12 enriched with 10% fetal bovine serum. These dishes were maintained at 37 °C in a 5% CO2 humidified incubator. The culture medium was routinely changed to remove floating cells. Once the cells reached confluence, they were subjected to trypsinization using 0.25% trypsin-EDTA for 2-3 minutes at room temperature to detach the less adherent cells, which included primary non-CAFs cells such as GBM cells, macrophages, and endothelial cells, as confirmed by microscopy. The highly adherent CAFs were then scraped and transferred to a new plate at a dilution of ½. This process of trypsinization following confluence, along with the scraping of the CAFs, was repeated at least three times to achieve a uniform population exhibiting a fibroblastic morphology.

***Immunofluorescence Staining.*** Cells seeded in Lab-Tek chamber slides were fixed with 4% PFA for 15 min at RT. Quenching and permeabilization steps were performed using PBS solution containing 5% BSA and 0.3% Triton-X100. Primary antibodies were incubated overnight at 4°C in PBS 5% BSA and 0.3% Triton-X100 solution. Secondary antibodies were incubated in PBS 5% BSA and 0.3% triton-X100 for 1 hour at room temperature. Mounting was performed with ProLong antifade Mounting Medium. Immunofluorescence stainings were analyzed under a microscope EVOS™ M5000 Imaging System (Invitrogen).

***RNA extraction, Reverse Transcription, and Real-time PCR* .** RNA was extracted using the RNeasy Plus Micro Kit from Qiagen, followed by reverse transcription using the PrimeScript RT Reagent kit from TAKARA. Real-time PCR was conducted with the ABI-Stepone+ system from Applied Biosystems, employing GAPDH for normalization. The compilation of primers employed to amplify the various target genes is available in the in the following Table.

**Table:** 

**Genes**	**Primers sequences**
ACTA2	S : CCCCATCTATGAGGGCTATGC AS : CGGCCAGCCAGATCCA
FAP	S : TCCAGTCTCCAGCTGGGAAT AS : GTTGGGAGACCCATGAATCTCT
LOX	S : AGGCCACAAAGCAAGTTTCTG AS : AAATCGCCTGTGGTAGCCATA
COL5A1	S : CCCGGATGTCGCTTACAGA AS : GAAATGCAGACGCAGGGTACA
COL6A1	S : GCGACGCACTCAAAAGCA AS : CTTGATAGCGCAGTCGGTGTAG
CD45	S : TCCAAGAGGAAAGACTCTCGAACT AS : GCAGGAAGCTGCTCCACACT
AIF	S : GGATGATGCTGGGCAAGAGA AS : CGCTTTTTCCTCATACATCAGGAT
PECAM1	S : TCTCCCAGCCCAGGATTTC AS : TTCGATGGTCTGTCCTTTTATGAC
EPCAM	S : TTGCCGCAGCTCAGGAA AS : AAAGCAGTTTACGGCCAGCTT
GFAP	S : CCGCAGCCCTGAAAGAGA AS : TTGCTGGACGCCATTGC
SOX2	S: TGCGAGCGCTGCACAT AS: TCATGAGCGTCTTGGTTTTCC
IL6	S : GCTGCAGGCACAGAACCA AS : GCTGCGCAGAATGAGATGAG
CXCL1	S : CCACTGCGCCCAAACC AS : GCAAGCTTTCCGCCCATT
CXCL6	S : GCAGTGCTCCAAGGTGGAA AS : CGGGTCCAGACAAACTTGCT
GAPDH	S : TGCACCACCAACTGCTTAGC AS : GGCATGGACTGTGGTCATGAG

***Cells irradiation and survival rate.*** CAFs were exposed to different doses of IR (4 or 6 Gy) using the SmART+ irradiator (Precision X-ray Inc., Madison, USA). The number of cells was quantified at specified time points, as indicated, using the Countess II FL cell counter.

***Statistical analysis.*** Unpaired T-tests were used to compare two groups. One-way ANOVA tests were performed for multiple comparisons.

## Reagents

**Table:** 

**Reagent**	**Source**
Dulbecco's Modified Eagle Medium (DMEM) F12	SIGMA D8437
anti-α-SMA-CY3 antibody	SIGMA C6198
anti-GFAP antibody	SIGMA AB5804
Anti-AIF1 antibody	Thermo Fisher Scientific PA5-27436
Secondary antibody anti-Rabbit IgG Alexa Fluor 488	InVitrogen A21206
Secondary antibody ant-Mouse IgG Alexa Fluor 494	InVitrogen A11032
RNeasy Plus Micro kit	QIAGEN 74034
PrimeScript RT Reagent Kit	TAKARA
ProLong Gold Antifade	Thermo Fisher Scientific P36931
